# Workforce Contributions to Advancing Oral Health Equity: Howard University’s Orthodontic Program

**DOI:** 10.3390/dj14030144

**Published:** 2026-03-05

**Authors:** Racine Ramanand, Kathy Marshall, Minxuan Lan, Marzia Mustamand, Bao Vu, Lobat Zainali, Marianne Siewe, Andrea D. Jackson, Indra Mustapha, Xinbin Gu

**Affiliations:** 1 College of Dentistry, Howard University, Washington, DC 20059, USA; 2 Department of Geography and Planning, The University of Toledo, Toledo, OH 43606, USA

**Keywords:** public health, orthodontics, workforce diversity, oral health disparities, health disparities, workforce representation

## Abstract

**Background**: Health disparities are differences in healthcare access influenced by factors such as race, socioeconomic status, and geography. Oral health disparities show similar patterns, with underrepresented minorities (URM) facing greater barriers to care. Racial concordance improves patient outcomes, yet minority representation in orthodontics remains low. This study examined how Howard University’s Orthodontic Residency Program influences workforce diversity and expands care for minority populations. **Methods**: A retrospective analysis of Howard University’s orthodontic graduates (Classes 2009–2024) was performed to collect demographic information, practice location, and board certification status. Practice addresses were evaluated for Health Professional Shortage Area (HPSA) designation, and geographic analysis identified the demographics of the practice areas. **Results**: Among the 94 graduates studied, thirty-seven (39.4%) worked in the surrounding Washington, D.C., Maryland, and Virginia (DMV) area. Currently, 30% of graduates practiced in HPSAs, and 53% practiced in majority-minority communities. Board certification rates showed an upward trend, culminating in 100% certification among 2024 graduates. **Conclusions**: Howard University’s Orthodontic Program has significantly advanced access to specialized care in majority-minority communities through the training of URM orthodontists. Alumni demographics, board certification rates, and placement in underserved areas demonstrate the program’s success in developing diverse professionals committed to clinical excellence and service.

## 1. Introduction

According to the World Health Organization (WHO), social determinants of health are defined as the circumstances under which persons are born, develop, live, and age, and their consequent implications [1,2]. Examples of social determinants of health include job status, income, educational attainment, gender inequality, and racial inequality [1,2]. Variation in these key determinants can lead to inequitable access to healthcare, which is defined as a health disparity [3]. These health disparities are often linked to social, economic, and environmental disadvantages, and they disproportionately affect individuals based on factors such as race/ethnicity, degree of education, income level, and geographic location [3].

Oral health disparities are a subset of health disparities, which are specific to differences in access to dental care and treatment outcomes [4]. Populations affected by oral health disparities often experience higher rates of oral diseases, such as tooth decay and gum disease, and may have limited access to preventive and restorative dental treatments [4]. Consistent with broader patterns of health disparities, limited access to dental care is closely associated with socioeconomic status, insurance coverage, racial/ethnic identity, and oral health literacy [4]. The breadth of literature demonstrates that numerous complex and interconnected factors influence access to oral healthcare; however, the tumultuous history of the United States points to race being one of the common denominators [5].

Within the healthcare context, racial concordance describes shared racial or ethnic identity between a patient and their provider [6]. Racial concordance has many advantages, as it has the potential to foster improved patient–provider communication, increase patient comfort, reduce the likelihood of medical mistrust, improve patient satisfaction, and even potentially improve treatment outcomes [6,7,8]. However, achieving greater racial concordance requires a proportional increase in diversity across healthcare professions [9]. Expanding diversity in the healthcare workforce has the potential to mitigate oral health disparities, in part by increasing the likelihood that minority providers practice in underserved communities and by incentivizing minorities to seek healthcare through the phenomenon of racial concordance, a factor shown to improve healthcare utilization among minority populations [10].

Though disparities in access to orthodontic care are less commonly discussed, unfortunately, the trends in this specialty are no different [11,12,13]. In fact, representation of minority groups is even lower in the orthodontic workforce than in the field of general dentistry, and it is no coincidence that Black and Hispanic children have significantly lower odds of an orthodontic visit than White children [14,15]. According to the U.S. Department of Labor Occupational Outlook Handbook, while there are an estimated 9500 practicing Orthodontists in the United States, only a reported 3.1% (295) are African American, approximately 1 in 32, while African Americans make up 12.4% of the U.S. population, approximately 1 in 8 individuals [16,17].

In addition to workforce diversity, access to orthodontic care is shaped by structural and policy-level factors, especially provider participation in Medicaid [18]. Prior research has consistently demonstrated that low reimbursement rates and administrative burdens reduce dental provider participation in Medicaid, thereby limiting access to care for publicly insured populations, even when coverage is available [18,19]. Reduced participation has been associated with lower utilization of dental services among Medicaid beneficiaries, highlighting that insurance coverage alone does not ensure access to care [18,19]. These barriers are further compounded in geographically underserved areas, such as Health Professional Shortage Areas (HPSAs), where existing workforce shortages intensify the impact of limited Medicaid participation [20,21]. Consequently, evaluating the orthodontist distribution of orthodontists within HPSAs provides critical context for understanding how workforce availability and systemic policy barriers intersect to influence access to orthodontic care for underserved populations [18,21].

Howard University’s Orthodontic Residency program is one of the foremost producers of minority orthodontists in the U.S. [22]. Established in 1967, the program has produced more than 300 orthodontists. One of the core tenets of Howard University’s orthodontic program is to train and prepare clinicians to serve communities where healthcare disparities persist [22]. This study seeks to evaluate the contributions of minority healthcare providers, particularly orthodontists trained through Howard University’s (HU) Orthodontic Residency program. This retrospective analysis focuses on their role in strengthening the orthodontic workforce by bridging the gaps in care and mitigating healthcare disparities in communities nationwide. This analysis will be based on the current practice locations of graduates from Howard University College of Dentistry’s Orthodontic Residency program between the years 2009 and 2024.

## 2. Materials and Methods

### 2.1. Data Compilation Procedure

This retrospective study assessed how Howard University-trained orthodontists contribute to closing gaps in access to orthodontic care by analyzing the geographic distribution of their practice locations. An inventory of Howard University-trained orthodontists graduating between 2009 and 2024 was obtained, and all verified graduates from this period were included in the analysis. Data collected included gender, race/ethnicity, contact information, current practice address(es), including ZIP code(s), and board certification status.

Practice location information was obtained from publicly available sources and cross-verified. Graduates for whom practice location data could not be confidently confirmed were excluded from the analysis to minimize potential misclassification. Each confirmed practice address was then evaluated using the Health Resources and Services Administration (HRSA) database to determine whether it was located within a dental Health Professional Shortage Area (HPSA), thereby classifying the practice as serving an underserved area.

Orthodontists with multiple verified practice locations were analyzed at the provider level. Individuals were classified as practicing in a dental Health Professional Shortage Area (HPSA) if at least one confirmed practice address was located within an HPSA. Each orthodontist was counted once in the analysis, regardless of the total number of practice sites, to prevent an overrepresentation of providers with multiple locations. Only confirmed graduates working within an HPSA were included. For practices spanning multiple ZIP codes, a graduate was counted as practicing in an HPSA if any portion of their practice fell within a designated shortage area. There are currently no publicly available datasets that report the number or proportion of orthodontists practicing within dental Health Professional Shortage Areas (HPSAs), preventing statistical comparisons to national benchmarks.

Geographic Information System (GIS) mapping was employed to provide insights into the demographic data and average income of the local population. GIS analysis was performed using ZIP codes of orthodontic practices founded by Howard University graduates to identify spatial clustering, evaluate proximity to designated underserved communities, and depict geographic distribution patterns.

The study was reviewed and granted exempt status by the Howard University Institutional Review Board (IRB-2025-1688, approval date 6 October 2025) in accordance with 45 CFR 46.101(b)(4).

### 2.2. Data Collection Platforms

*Provider Identity and Practice Information:* The National Provider Identifier (NPI) Registry (https://npiregistry.cms.hhs.gov/ (accessed on 12 October 2025) was utilized to confirm provider identity and to validate the location and address of their clinical practice. Additional professional and biographical information was obtained through publicly accessible online resources, including Google search results and LinkedIn profiles.

*Underserved Area Classification:* Practice locations were evaluated against federal designations of MUAs and Health Professional Shortage Areas (HPSAs) using data from Health Resources and Services Administration (HRSA) (https://data.hrsa.gov/ (accessed on 12 October 2025).

## 3. Results

### 3.1. Study Sample

The study involved 94 subjects from Howard University College of Dentistry’s (HUCD) Orthodontic Residency program graduating classes from 2009 to 2024. Of these, four alumni were practicing internationally, while the remaining 90 were practicing within the United States. The four international alumni practicing in countries such as Canada, Kuwait, and Jamaica were excluded from the geographical analysis.

### 3.2. Graduate Demographic and Representation

**Figure 1:**
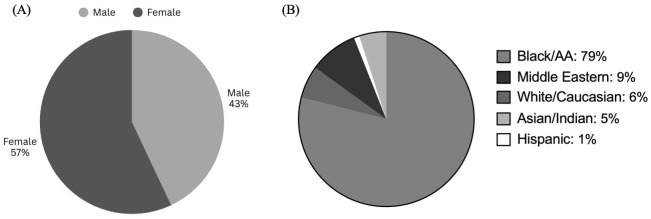
Gender and Racial/Ethnic Composition of HU-Trained Orthodontists (Classes 2009–2024): (**A**) Female-to-male enrollment ratio at Howard University’s Orthodontic Residency Program over 15 years. (**B**) Racial and ethnic makeup of HU-Trained Orthodontists during the same period.

The gender distribution of graduates of the Howard University (HU) orthodontic program (2009–2024) showed that 57% percent of Howard University-trained orthodontists self-identified as female and 43% self-identified as male (Figure 1A). This demonstrates that a higher proportion of female orthodontic graduates compared to male orthodontic graduates, thereby indicating a slight female prevalence. The racial/ethnic distribution of residents at HU’s orthodontic program (2009–2024) indicates the program’s emphasis on training minority healthcare providers. During this period, 79% of residents self-identified as Black/African American, 6% as White/Caucasian, 9% as Middle Eastern, 5% as Asian/Indian, and 1% as Hispanic (Figure 1B). The predominance of Black/African American graduates underscores Howard University’s role as a leading institution in bridging gaps in access to culturally competent and diverse orthodontic care.

### 3.3. Overall Graduate Placement

**Figure 2:**
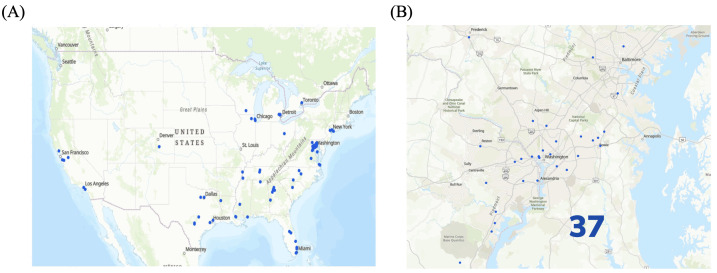
Geographic Distribution and Demographic Composition of HU-Trained Orthodontists (Classes 2009–2024): (**A**) Map of the United States showing the location distribution of Howard University (HU)-trained orthodontists. (**B**) Map of the District of Columbia, Maryland, and Virginia (DMV) area showing the location distribution of Howard University-trained orthodontists.

Graduates of HU’s orthodontic program (2009–2024) have established practices widely across the United States and provide services to a broad range of geographic areas (Figure 2A). Alumni were distributed across numerous states and regions, including the Northeast, South, Southwest, and West Coasts, reflecting a national reach, with the highest concentration of graduates remaining in the District of Columbia, Maryland, and Virginia (DMV) area (Figure 2A,B). This widespread distribution demonstrates the program’s impact in expanding access to orthodontic care across diverse communities. Specifically, 37 out of the 94 (39%) total residents chose to practice orthodontics within the DMV area (Figure 2B). These data indicate that Howard University has played a significant role in diversifying the orthodontic workforce in the DMV region over the last 15 years.

### 3.4. Practice in Health Professional Shortage Areas and Board Certification Status Among HU-Trained Orthodontic Graduates

**Figure 3:**
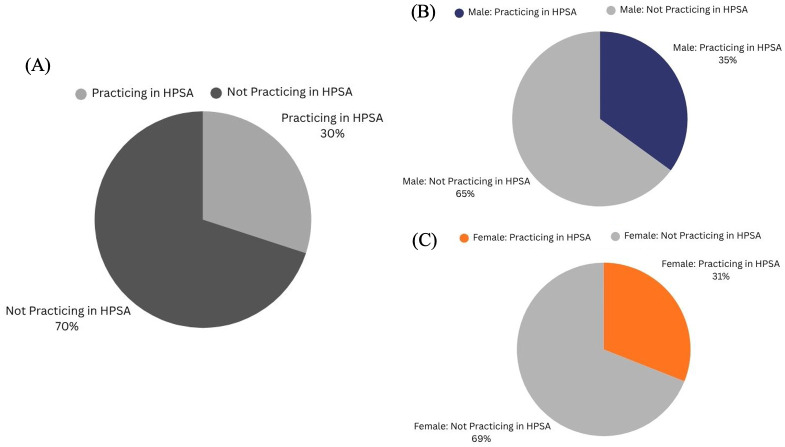
Howard University (HU)-Trained Orthodontists HPSA Status (Classes 2009–2024): (**A**) Overall comparison of HU-trained orthodontists practicing in designated Health Professional Shortage Areas (HPSAs) (Classes 2009–2024). (**B**) Comparison of the proportion of male HU-trained orthodontists practicing in designated Health Professional Shortage Areas (HPSAs). (**C**) Comparison of the proportion of female HU-trained orthodontists practicing in designated Health Professional Shortage Areas (HPSAs).

**Figure 4:**
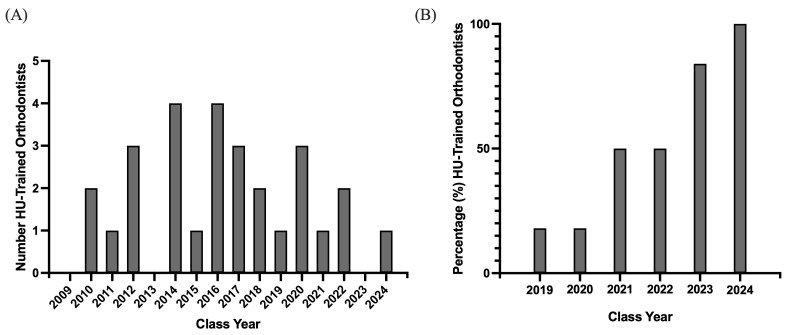
Howard University (HU)-Trained Orthodontists Practicing Dental HPSA and Board-Certification Status: (**A**) Bar graph depicting temporal trends (2009–2024) in the number of Howard University-trained orthodontists currently practicing in federally designated Dental Health Professional Shortage Areas (HPSAs). (**B**) Bar graph depicting the percentage of Howard University-trained orthodontists (2019–2024) who have achieved board certification through the American Board of Orthodontics (ABO).

Between 2009 and 2024, 30% of HU’s orthodontic program graduates established practices in HRSA-designated Health Professional Shortage Areas, whereas the remaining 70% practiced in non-HPSA locations (Figure 3A). This distribution indicates that nearly a third of the program’s alumni are contributing to providing dental care in underserved communities. While the proportion of HU’s orthodontic program graduates (2009–2024) practicing in Health Professional Shortage Areas (HPSAs) appeared to be modest, the data overall showed no consistent trend over time, suggesting that the decision to practice in an HPSA was likely influenced by individual factors (Figure 4A).

Distinctively, male graduates were slightly more likely to practice in HPSAs compared to their female counterparts (Figure 3B,C). Specifically, 35% of male graduates practiced in HPSAs, compared to 31% of female graduates (Figure 3B,C). Although the difference was minor, these findings suggest a relatively balanced distribution of service to underserved areas across genders, with a slightly greater tendency for male graduates to practice in shortage areas.

Prior cohorts in the study group (2009–2018) demonstrated inconsistent and highly variable patterns in board certification rates. These years were therefore excluded from the analysis due to the absence of a discernible or interpretable trend. Notably, recent years have shown significant increases in certification rates, culminating in 100% board certification among the 2024 graduating class (Figure 4B). Beginning in 2021, there was a prominent increase, with 50% of graduates certified in both 2021 and 2022, 83% in 2023, and culminating in 100% board certification among the 2024 graduates (Figure 4B).

### 3.5. Median Household Income in Communities Served by HU-Trained Orthodontists

**Figure 5:**
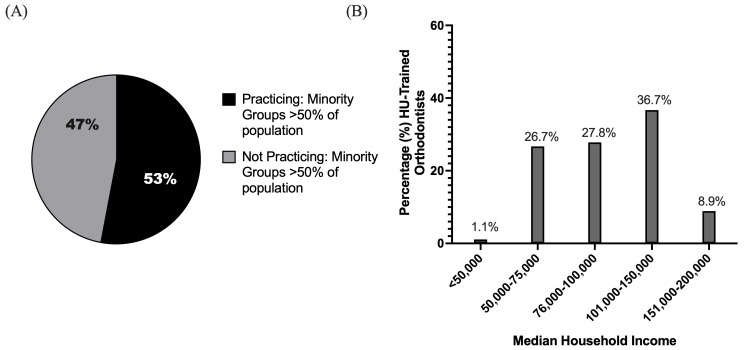
Practice Community Characteristics of Howard University (HU)-trained orthodontists (Classes 2009–2024): (**A**) Distribution of HU-trained orthodontists practicing in communities where racial and ethnic minority populations constitute the majority. (**B**) Bar graph illustrating the distribution of Howard University-trained orthodontists (2009–2024) by the median household income level of the communities in which they currently practice.

Graduates of HU’s orthodontic program (2009–2024) currently practice in regions spanning varying median household income levels (Figure 5B). Of the alumni analyzed, 36.7% practiced in communities with a median income between $101,000 and $150,000, 27.8% in areas with median incomes between $76,000 and $100,000, 26.7% in areas with median incomes between $50,000 and $75,000, 8.9% in areas with median incomes greater than $150,000, and 1.1% practicing in areas with median incomes less than $50,000 (Figure 5B).

An analysis of the practice locations of graduates from HU’s orthodontic program (2009–2024) revealed that 53% of graduates currently practiced in areas where minority groups comprise the majority of the population, identified as locations in which the Caucasian/White population constitutes less than 50% of the area’s total population (Figure 5A). The remaining 47% practiced in areas where minority populations do not represent the majority (Figure 5A). This distribution highlights that more than half of the Howard-trained orthodontists are serving communities with significant minority populations, aligning with the program’s broader mission to address healthcare disparities and improve access to orthodontic care for historically underserved groups.

## 4. Discussion

Howard University’s Orthodontic Residency Program is one of the foremost producers of minority orthodontists in the U.S. Established in 1967, in its 58-year history, the program has produced more than 300 minority orthodontists [22]. Howard University College of Dentistry’s (HUCD) orthodontics program is distinguished by its dedication to addressing and eliminating disparities in oral healthcare [22]. This study sought to assess the influence of HU-trained orthodontists on the oral health workforce while also examining the persistent barriers that prevent improved access to orthodontic care. Analyzing trends among HU’s orthodontic program graduates revealed important patterns in gender distribution, racial and ethnic representation, practice locations in shortage areas, and board certification status. Our findings highlight the program’s enduring role in addressing oral health disparities and promoting orthodontic care for underserved populations through diversity-focused recruitment and service-centered training.

### 4.1. Advancing Workforce Diversity Through the Demographic Landscape

The gender distribution data demonstrated a sustained increase in female representation, with 57% of graduates being identified as female. Interestingly, this trend also mirrors national shifts in dental and orthodontic education, where women have steadily increased their presence. The racial and ethnic composition of graduates strongly reflected HUCD’s founding mission: to promote diversity in oral healthcare. With 79% of Orthodontic Residency alumni from 2009 to 2024 identified as Black/African American, Howard University plays a critical role in training minority orthodontists. This is particularly impactful given that African Americans represent only 3.1% of practicing orthodontists nationally, despite comprising 12.4% of the U.S. population. Increasing minority representation in orthodontics addresses barriers to care by fostering racial concordance, which has been shown to improve patient trust, satisfaction, and clinical outcomes among patients and providers of the same race(s).

### 4.2. Geospatial Patterns and Healthcare Access Challenges

Geographic analysis trends demonstrated that HU-trained orthodontists are broadly distributed across all regions of the United States, with four in international locations such as Canada, Kuwait, and Jamaica, yet nearly 40% have chosen to remain in the Washington, DC, Maryland, and Virginia (DMV) area. This reflects HU’s deep community ties as well as its importance in instilling the values of philanthropy into its residents, aiming to improve orthodontic access in an area with persistent healthcare disparities. While trends in HPSA practice were inconsistent over time, male graduates were slightly more likely to practice in shortage areas (35% of males and 31% of females). This difference may be influenced by career opportunity structures, personal motivations related to service, perceived safety, or geographic preferences.

Of particular significance, over half (53%) of the alumni from 2009–2024 are currently practicing in communities where minority groups constitute the majority of the population. This indicates that Howard-trained orthodontists are not only contributing to the dental workforce but are also strategically positioned to serve populations disproportionately affected by barriers to orthodontic care. The analysis revealed that 30% of HU-trained orthodontists elected to practice within designated Health Professional Shortage Areas (HPSAs). Socioeconomic analysis of practice locations indicated that while 36.7% of graduates practiced in communities with median incomes between $101,000 and $150,000, a notable 26.7% practiced in communities with median incomes between $50,000 and $75,000.

Although these proportions may appear modest, they must be interpreted within the broader structural context in which access to orthodontic care is constrained by low provider participation in Medicaid for such services [23]. A study conducted in North Carolina found that low fee reimbursement was consistently cited as a major barrier to participation, limiting access for Medicaid-eligible patients [23]. Comprehensive studies such as these provide a framework to interpret our results and highlight the importance of healthcare policy reform to expand access to orthodontic care among underserved populations. These systemic limitations disproportionately affect underserved communities; therefore, even a relatively small increase in the number of orthodontists in HPSAs may yield a substantive improvement in care accessibility and contribute to mitigating existing disparities in oral health outcomes [24,25].

Moreover, the current literature provides limited evidence regarding whether other orthodontic training institutions demonstrate higher rates of graduate placement in HPSAs, making it difficult to contextualize these findings within broader national trends. Dental education programs similar to HU’s, with a mission focused on serving underserved or rural communities, generally report higher proportions of graduates practicing in these areas. For example, graduates of a dental training program with an explicit rural and underserved mission were reported to practice in underserved or rural communities at a rate of approximately 78% [26]. However, to our knowledge, there are no published benchmarks specifically for orthodontic residency programs, and direct comparisons to our observed 30% HPSA placement rate are therefore not possible. As such, the implications of this placement rate remain uncertain and warrant further investigation.

Additionally, we did not investigate the factors influencing each graduate’s practice location decisions. However, prior research on dental and medical workforce patterns suggests that such decisions are often influenced by individual factors, including personal preference, financial considerations, family or lifestyle priorities, and perceived safety [27]. It is therefore plausible that similar individual-level factors contributed to the observed distribution of HU graduates.

Notably, longitudinal analysis revealed a clear upward trajectory in ABO board certification for HU orthodontic graduates, culminating in 100% certification among the 2024 class. Prior years (2004–2018) of the study group saw inconsistent trends in board certification. The steady rise beginning around 2021 reflects both changes within the residency program itself, encouraging a higher standard of excellence, as well as changes in the ABO certification process that occurred in 2020 in response to the COVID-19 pandemic [28]. The change from an oral format to a written scenario-based format has removed barriers to and greatly incentivized achieving board certification, as it is now more convenient than ever [28]. This upward trend indicates meaningful progress in aligning the orthodontic training at Howard University with national standards.

The discussion of racial and socioeconomic inequities in orthodontic care is both important and underexplored. Disparities in access and utilization mirror broader oral healthcare inequities, with evidence showing that race and socioeconomic status significantly influence who receives treatment and how often [12]. National data indicate that Black and Hispanic children undergo fewer orthodontic procedures than White children, even after accounting for insurance and income [12]. Longitudinal research further shows that non-Hispanic Black and Hispanic individuals are more likely to follow low or declining dental care trajectories compared with non-Hispanic Whites, highlighting persistent barriers to consistent care [29]. These findings reinforce that structural and individual factors continue to drive inequitable access to orthodontic and general oral healthcare [30].

Within this context, Howard University’s Orthodontic Residency Program represents a tangible approach to addressing these persistent disparities. By training predominantly minority orthodontists, the program increases access to specialized care in majority-minority communities while fostering clinical excellence, social responsibility, and culturally concordant care. Such initiatives offer a model for other dental education programs aiming to promote a more equitable healthcare system.

### 4.3. Limitations and Future Directions

Despite verification efforts, some degree of misclassification may persist due to reliance on publicly available data sources. The limitations of this study include the possibility of obtaining outdated information, such as previous practice locations of Howard-trained orthodontists. Similarly, if a graduate recently changed practice locations, this update may not yet be reflected publicly. National comparisons with graduates from other minority-focused dental or orthodontic programs were not performed because, to our knowledge, no publicly available datasets report demographic or practice location outcomes specifically for orthodontic graduates by HPSA status or by minority-focused program participation. Existing national workforce data indicate an under-representation of URM dentists in specialty training, including orthodontics, but the absence of linked specialty-specific demographic workforce data precludes formal statistical benchmarking [11].

Future research should aim to include a comprehensive, longitudinal study encompassing the 58-year history of the HU’s orthodontic program and over 300 of its trained orthodontists. This would provide an even more comprehensive view of long-term trends in practice locations, particularly regarding service in HPSAs and board certification rates. Furthermore, conducting comparable studies at other orthodontic residency programs would help establish benchmarks to contextualize HUCD’s 30% HPSA placement rate. This contextualization would clarify whether the trends among Howard-trained orthodontists reflect the university’s distinct culture and mission, or if comparable patterns occur across other programs irrespective of institutional demographics or objectives.

## 5. Conclusions

HU’s orthodontic program has made an immense impact through its continued efforts in training predominantly minority orthodontists. The program has helped expand access to specialized care in communities where minority populations constitute more than half of the residents. Rising board certification rates, alumni demographics, and the proportion practicing in majority-minority communities collectively highlight the program’s success in developing diverse leaders dedicated to clinical excellence and social responsibility. Continued support for initiatives that foster service and professional development will be essential to sustaining this progress. Ultimately, HUCD’s Orthodontic Residency Program serves as a model for how dental education programs can meaningfully contribute to a more equitable healthcare system.

## Data Availability

Study data were collected using publicly available websites: The National Provider Identifier (NPI) Registry (https://npiregistry.cms.hhs.gov/ (accessed on 12 October 2025) and Health Resources and Services Administration (HRSA) (https://data.hrsa.gov/ (accessed on 12 October 2025).

## Abbreviations

The following abbreviations are used in this manuscript:

URM	Underrepresented Minority
HPSA	Health Professional Shortage Area
DMV	Washington, D.C., Maryland, and Virginia
WHO	World Health Organization
HU	Howard University
HUCD	Howard University College of Dentistry
HRSA	Health Resources and Services Administration
GIS	Geographic Information System
NPI	National Provider Identifier
